# Interactions of genetic variations in *FAS*, *GJB2* and *PTPRN2* are associated with noise-induced hearing loss: a case-control study in China

**DOI:** 10.1186/s12920-023-01790-7

**Published:** 2024-01-11

**Authors:** Shan Wu, Zhidan Wu, Manlian Chen, Xiangbin Zhong, Haoyan Gu, Wenjing Du, Weidong Liu, Li Lang, Junyi Wang

**Affiliations:** 1 Guangdong Provincial Engineering Research Center of Public Health Detection and Assessment, School of public health, Guangdong Pharmaceutical University, Guangzhou, China; 2 Guangzhou Baiyun District Center for Disease Prevention and Control, Guangzhou, China; 3 The Sixth people’s Hospital Of Dongguan, Dongguan, China; 4 Guangdong Province Hospital for Occupational Disease Prevention and Treatment, Guangzhou, China

**Keywords:** Genetic susceptibility, Occupational noise-induced hearing loss (NIHL), Single nucleotide polymorphism (SNP), Genetic risk score (GRS), Classification and regression tree (CART)

## Abstract

**Background:**

This study aimed to screen and validate noise-induced hearing loss (NIHL) associated single nucleotide polymorphisms (SNPs), construct genetic risk prediction models, and evaluate higher-order gene-gene, gene-environment interactions for NIHL in Chinese population.

**Methods:**

First, 83 cases and 83 controls were recruited and 60 candidate SNPs were genotyped. Then SNPs with promising results were validated in another case-control study (153 cases and 252 controls). NIHL-associated SNPs were identified by logistic regression analysis, and a genetic risk model was constructed based on the genetic risk score (GRS), and classification and regression tree (CART) analysis was used to evaluate interactions among gene-gene and gene-environment.

**Results:**

Six SNPs in five genes were significantly associated with NIHL risk (*p* < 0.05). A positive dose-response relationship was found between GRS values and NIHL risk. CART analysis indicated that strongest interaction was among subjects with age ≥ 45 years and cumulative noise exposure ≥ 95 [dB(A)·years], without personal protective equipment, and carried *GJB2* rs3751385 (AA/AB) and *FAS* rs1468063 (AA/AB) (OR = 10.038, 95% CI = 2.770, 47.792), compared with the referent group. *CDH23*, *FAS*, *GJB2*, *PTPRN2* and *SIK3* may be NIHL susceptibility genes.

**Conclusion:**

GRS values may be utilized in the evaluation of the cumulative effect of genetic risk for NIHL based on NIHL-associated SNPs. Gene-gene, gene-environment interaction patterns play an important role in the incidence of NIHL.

**Supplementary Information:**

The online version contains supplementary material available at 10.1186/s12920-023-01790-7.

## Introduction

The candidate-gene approach isdriven by hypothesis based on the prior knowledge of single nucleotide polymorphisms (SNPs) and gene functions, and has often produced informative but sometimes contraditory results in studies related to hearing loss. In many studies that reported significant correlations, the odds ratios (ORs) of individual variants were < 2 [1, 2]. The low risk induced by individual polymorphism is not surprising, because the occurrence of hearing loss is usually a multi-step, multigenic process, and any single genetic polymorphism is unlikely to have a dramatic impact on hearing loss risk. Thus, a single gene study may have limited power to predict risk. However, it is unkonwn whether genetic predisposition to hearing loss expansion is due to the interaction of multiple susceptibility genes. Multigenic approachs, which evaluated the joint effects of multiple polymorphisms, may enlarge the influence of individual polymorphisms and enhance the predictive ability. Several recent multigenic studies have demonstrated the promising potential of using such a multigenic approach in correlation studies [3, 4]. In this study, we use noise-induced hearing loss (NIHL) as the hearing loss prototype to illustrate our theme.

NIHL is one of the most widespread prevalent occupational disease, and the second most common sensorineural hearing loss following age-related hearing impairment [5]. NIHL is a polygenic disease induced by the interaction of environmental and genetic factors. The known environmental factors such as noise exposure, organic solvent exposure, smoking and drinking are responsible to NIHL [6, 7]. It was reported that individuals represent variable degrees of NIHL susceptibility, even when exposed to the same levels of noise intensity [8]. Human studies have revealed that the heritability of NIHL in twins is approximately 36% [9]. Knockout mice, such as *CDH23*^+/−^ [10], *GPX1*^−/−^ [11], *PMCA2*^−/−^ [12], *SOD1*^−/−^ [13], were more susceptible to noise than their wild-type mice. Previous studies have discovered that more than one hundred SNPs may be related to the NIHL susceptibility, including heat shock proteins genes, oxidative stress genes, cadherin proteins genes, potassium recycling channel genes, apoptosis signaling genes, calcium ions recycling channel genes, and inflammatory factor genes [8, 14]. However, there are countless examples in which correlation studies have failed to replicate positive candidate-gene findings, which may be due to small sample size, insufficient statistical methods, and failure to assess the joint effect of multiple pathophysiological associated genes. Therefore, these findings are not enough to explain the total heritability of NIHL.

In present study, we used a multigenic approach to estimate the relationships of gene polymorphisms with NIHL risk. We performed a two-stage case-control study to screen and validate NIHL-associated SNPs among the Chinese populations. As far as we know, this is the most comprehensive multigenic NIHL correlation study reported. We also built a genetic risk score (GRS) model based on these NIHL-related SNPs to better capture the joint effects of multiple genes. Besides, we applied classification and regression tree (CART) to investigate high-order gene-gene, gene-environment interactions in NIHL susceptibility. Our findings will expand our understanding of the genetic basis of NIHL.

## Materials and methods

### Study participants

**Fig. 1:**
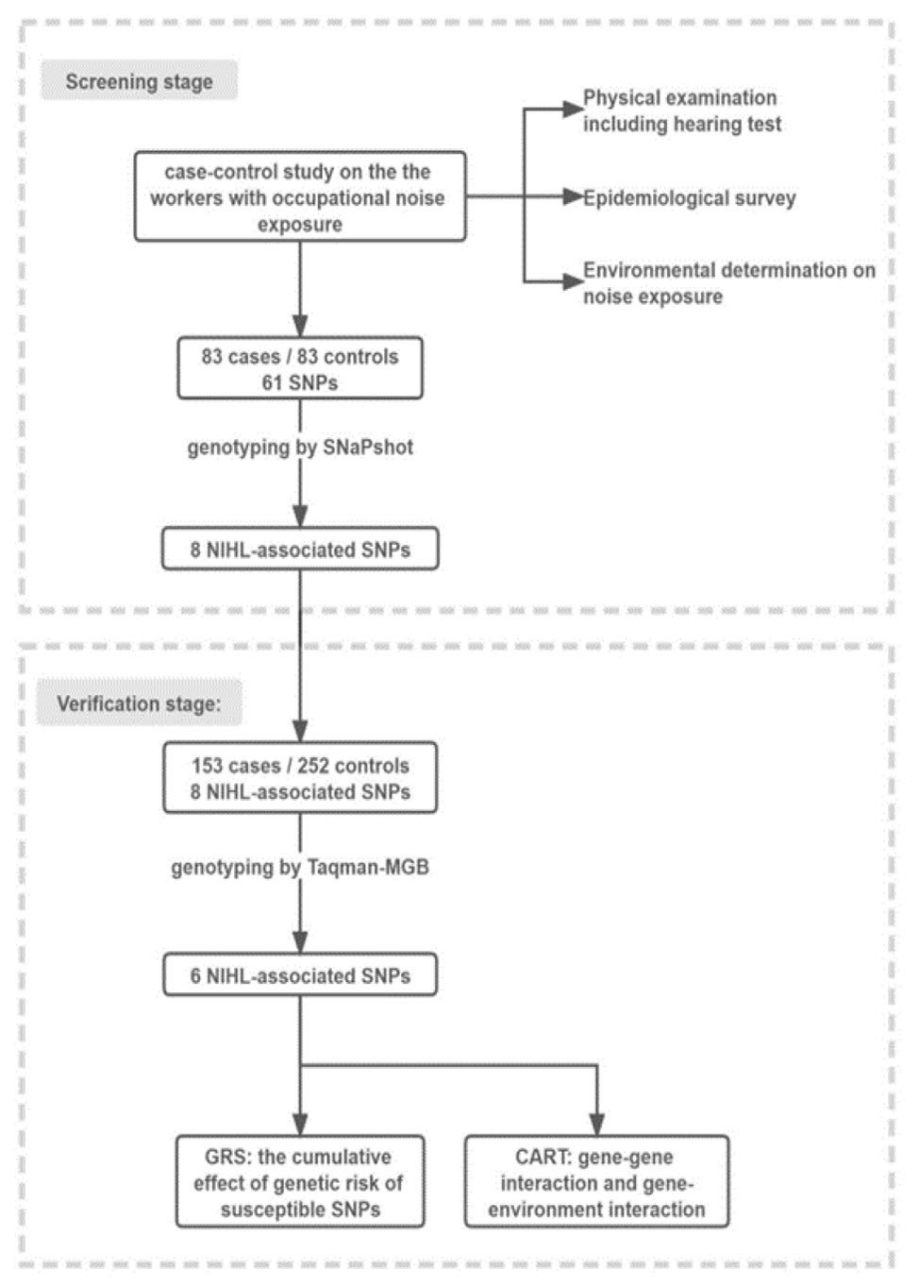
The subjects were recruited from the workers with occupational noise exposure. 83 NIHL subjects and 83 controls were genotyped. Then NIHL-associated SNPs were validated in another case-control study (153 cases and 252 controls), and GRS model and CART analysis were constructed

We conducted a two-stage case-control study to screen and validate NIHL-associated SNPs among the Chinese populations. The flow chart of this study is presented in Fig. 1. All participants were enrolled from the people who underwent NIHL diagnosis and occupational physical examination from September 2019 to October 2021 in Guangdong Province Hospital for Occupational Disease Prevention and Treatment and the Sixth People’s Hospital of Dongguan. The noise exposed worker was defined as the worker working in the workplace that exceed the occupational noise exposure limit, that is, eight-hour continuous equivalent dB(A) weighted sound pressure levels (L_Aeq, 8 h_) ≥ 85 dB(A). According to the Diagnostic Standards for Occupational Noise-induced Deafness of the People’s Republic of China (GBZ 49-2014) (China, 2014), participants diagnosed with occupational noise-induced deafness were included in the NIHL group. The hearing evaluation of subject was based on the results of air-conduction pure tone audiometry (PTA). NIHL group was diagnosed by the binaural high frequency threshold average (BHFTA) (3000 Hz, 4000 Hz, 6000 Hz), the monaural threshold of weighted value (MTWV) calculated by 90% low frequency (500 Hz, 1000 Hz, 2000 Hz) hearing threshold mean weighting and 10% high frequency (4000 Hz) hearing threshold. NIHL was defined as follows: the noise exposed workers with normal hearing before noise exposure, > 3 years of occupational noise exposure, the results of pure-tone air-conduction threshold pattern assessment were consistent with the trend of noise hearing impairment pattern characteristics, BHFTA were ≥ 40 dB, and MTWV were ≥ 26 dB. The inclusion criteria for the controls were as follows: > 3 years of occupational noise exposure, BHFTA were < 40 dB and MTWV were < 26 dB. All subjects had no hearing-related complications, ear trauma, otitis media, hereditary deafness and blast deafness, family history of hearing loss, craniocerebral injury and usage of certain drugs or toxins (such as aminoglycoside drugs, cisplatin, chloroquine). All the methods and procedures carried out in this study were in accordance with the Declaration of Helsinki. And this study was approved by the Science Ethics Committee of Guangdong Pharmaceutical University (20,190,212). All the participants signed the consent forms.

### Data collection

All subjects were interviewed face to face by a well-trained public health practitioner using a structured questionnaire that included general characteristics (sex, age, and ethnicity), factors related to noise exposure (factory, work situation, daily noise exposure duration, work years with noise exposure, use of personal protective equipment (PPE), exposure to other occupational harmful factors), and lifestyle factors (family history of hearing loss, medical history, medication use, smoking and drinking status).

Other information, including the results of hearing test and environmental noise measurement, was obtained from personal occupational health examination reports and occupational environment monitoring files. Hearing test was conducted by well-trained physicians according to standard procedures. After being out of the noise environment for at least 48 h, all participants underwent a PTA in a sound-insulation room with background noise less than 25 dB(A). The hearing threshold for each ear was measured at six frequencies, and the deviation of hearing threshold of each frequency was ≤ 10dB. Cumulative noise exposure (CNE) was calculated by years of noise exposure and sound pressure level (SPL) to assess individual noise exposure dosage. Based on the technical requirements of Workplace Physical Factors Measurement Part 8: Noise (GBZ/T189.8-2007) (China, 2007), environmental noise exposure level was assessed using L_Aeq,8 h_, the equivalent A-weighted sound pressure level of the contact noise intensity normalized to 8 h during the actual working time of a day. CNE is an evaluation parameter derived from the theory of equal energy exposure and equal biological effect, so that individual noise exposure levels of different noise exposure intensities and times have comparability.

### Blood collection and DNA extraction

Whole blood samples were collected from each participant after an overnight fasting. DNA was extracted from blood leucocytes using the TIANamp Genomic DNA Kit (centrifugation column method) (Tiangen, Bejing, China) following the manufacturer’s protocol. The integrity of DNA was measured by agarose gel electrophoresis, and the content was measured by a microspectrophotometer (NanoDrop 5000, Thermo Scientific, USA).

### SNPs selection and genotyping in the screening stage

83 NIHL cases and 83 controls were recruited. A total of 60 SNPs in 38 genes were selected as candidate SNPs according to the National Center for Biotechnology Information (NCBI) database and previous reports (Supplementary Table 1). The sequences of specific amplification primers and extension primers were designed on Primer Premier software (v6.0, Premier Biosoft Inc., CA). All the SNPs were genotyped on 3730XL gene sequencing instrument (Applied Biosystems Inc., USA) by the SNapShot assays at the Good Biotechnology Co., Ltd. (Guangzhou, China), and were analyzed with Gene Mapper software (v5.0, Premier Biosoft Inc., CA).

### SNPs verification in the replication stage

A total of 153 NIHL and 252 controls were selected. All the SNPs screened from the first stage were genotyped using the Taqman-MGB method. The design methods of TaqMan-MGB probe and primer were the same as above. Genomic DNA was added into 2 x TaqMan qPCR Master Mix (Vazyme Biotech Co., Ltd, Nanjing, China) with genotyping probes and primers according to the instructions of manufacturer, and the amplified products were scanned by fluorescence in the whole wavelength range on Real-time fluorescent quantitative PCR instrument (CFX96, Bio-rad, USA). Analyses were performed using BIORAD CFX Maestro software (v3.1, Bio-rad, USA).

### Statistical analysis

Continuous variables for the normal distribution were expressed as mean ± standard deviation (SD) and as median (P_25_, P_75_) for skewed distribution and analyzed by Student’s *t*-tests. Whether sample group was representative was decided by Hardy-Weinberg equilibrium (HWE) tests. Categorical variables were expressed as frequency (%) and analyzed by *χ*^2^ test. CNE = 10 × log (10^SPL^ * years of noise exposure), where SPL is the sound pressure level [dB(A)] of noise exposure. HWE was tested using Pearson’s *χ*^2^ for each SNP in the controls. SNPs were eliminated when the call rates were < 90% and the controls deviated from HWE with *p* < 0.01. Multiple logistic regression was conducted to calculate the OR and 95% confidence interval (CI) under the dominant, super-dominant, recessive, additive and allele model, and adjusted by confounders, including age, years of occupational noise exposure, smoking and drinking status.

To combine the relatively small effects of individual genes and to better explore the complex relationship between genetics and NIHL disease, GRS model was performed. The SNPs, whose *p* values < 0.05 in logistic regression model during the replication stage, were selected due to the limited sample size and false negative signals. GRS refers to the sum of risk genotypes (0 for non-risk genotypes, 1 for other genotypes, and 2 for risk genotypes) across NIHL-associated SNPs, and was calculated as reported previously [3]. The participants were divided based on the GRS values and the ORs were calculated with the subjects in the lowest levels of GRS (≤ 5) as a reference. The dose-response relationship between GRS value and OR was assessed using the *χ*^2^ test.

CART analysis was applied to establish a decision tree through recursive partitioning to determine specific combinations of genetic and environmental factors related to disease risk [15]. Based on the splitting rule of information index, the data were layered into individual subsets, which are represented as nodes in this decision tree. This process terminated when the classification achieves the lowest cross-validation error in the terminal nodes. Taking the lowest percentage of termination nodes as a reference, logistic regression analysis was conducted to calculate the OR value and its 95% CI of different branches, and adjusted by confounders such as age, years of occupational noise exposure, smoking and drinking status. The learning set was applied to build the tree model, and the testing set was used to internally verify the generated tree model. The data were randomly divided into a learning set (65% of the data) and a testing set (35% of the data).

All statistical analyses were conducted using R software (v 4.1.2). Two-tailed *p* < 0.05 indicated statistical significance, and 0.05 < *p* < 0.10 was marginal significance.

## Results

### Eight candidate SNPs were replicated in the screening stage

A total of 83 NIHL subjects and 83 controls were recruited (Supplementary Table 2). Briefly, the mean (SD) age, years of noise exposure and CNE was 48.32 ± 5.69 years, 12.24 ± 5.75 years and 96.51 ± 5.49 dB(A)·years, respectively. There was no statistical significance between the NIHL cases and controls regarding age, sex, ethnicity, years of noise exposure, CNE, use of PPE, smoking and drinking status (*p* > 0.05). The binaural high frequency threshold average (BHFTA) in NIHL group was 55.23 ± 10.38 dB, and was significantly higher than the control group (28.02 ± 7.49 dB; *p* < 0.05).

There were 15 SNPs with marginal or statistical significance by *χ*^2^ test. Seven SNPs in six genes (*CDH23* rs2394795, *FAS* rs1468063 and rs2862833, *GJB2* rs3751385, *HOTAIR* rs87494, *SIK3* rs6589574, *STAT3* rs1053005) were found to be significantly different between two groups under at least one genetic model (*p* < 0.05; Supplementary Table 3), and eight SNPs in seven genes (*FOXO3* rs2802292 and rs10457180, *GAPDH* rs6489721, *HDAC2* rs10499080, *hOGG1* rs1052133, *PTPRN2* rs10081191, *STAT3* rs1053023 and *TAB2* rs521845) were marginally significant (0.05 < *p* < 0.10; Supplementary Table 3). Finally, eight SNPs in six genes (*CDH23* rs2394795, *FAS* rs1468063 and rs2862833, *GJB2* rs3751385, *PTPRN2* rs10081191, *SIK3* rs6589574, and *STAT3* rs1053023 and rs1053005) were related to NIHL susceptibility in logistic regression model after adjusting for age, year of noise exposure, smoking and drinking status (*p* < 0.05; Table 1).

**Table 1:** Odd ratios (ORs) and 95% CI of NIHL-associated SNPs in the screening stage

Code	Gene	SNPs	Genotype	OR (95% CI)^*^	*p*	OR_adj_ (95% CI)^†^	*p*
1	*CDH23*	rs2394795					
		Recessive	AA + AB/BB	**2.190 (1.040, 4.789)**	**0.043**	**2.437 (1.133, 5.446)**	**0.025**
		Dominant	AA/BB + AB	0.705 (0.340, 1.467)	0.352	0.610 (0.272, 1.323)	0.216
		Super-dominant	AA + BB/AB	0.746 (0.402, 1.376)	0.349	0.752 (0.399, 1.411)	0.375
		Homozygote	BB/AA	2.423 (0.960, 6.317)	0.064^#^	**4.068 (1.383, 13.605)**	**0.015**
		The allele	B/A	1.472 (0.957, 2.273)	0.080^#^	**1.976 (1.148, 3.459)**	**0.015**
2	*FAS*	rs1468063					
		Recessive	BB/AA + AB	**2.456 (1.176, 5.341)**	**0.019**	**2.308 (1.090, 5.089)**	**0.032**
		Dominant	AA/BB + AB	0.896 (0.466, 1.717)	0.740	0.877 (0.445, 1.722)	0.704
		Super-dominant	AA + BB/AB	**2.101 (1.131, 3.951)**	**0.020**	**2.090 (1.100, 4.023)**	**0.026**
		Homozygote	AA/BB	1.857 (0.799, 4.442)	0.155	1.792 (0.742, 4.444)	0.199
		The allele	A/B	1.307 (0.848, 2.018)	0.226	1.382 (0.845, 2.272)	0.199
3	*FAS*	rs2862833					
		Recessive	BB/AA + AB	0.945 (0.487, 1.830)	0.866	0.934 (0.468, 1.862)	0.845
		Dominant	BB + AB/AA	**2.510 (1.223, 5.341)**	**0.014**	**2.437 (1.170, 5.274)**	**0.020**
		Super-dominant	AA + BB/AB	**1.899 (1.025, 3.558)**	**0.043**	1.884 (0.992, 3.617)	0.054^#^
		Homozygote	BB/AA	2.080 (0.903, 4.92)	0.089^#^	2.101 (0868, 5.247)	0.104
		The allele	B/A	1.438 (0.934, 2.221)	0.100	1.584 (0.965, 2.621)	0.071^#^
4	*FOXO3*	rs2802292					
		Recessive	BB/AA + AB	1.561 (0.536, 4.859)	0.420	1.427 (0.484, 4.497)	0.524
		Dominant	AA/BB + AB	0.643 (0.345, 1.188)	0.160	0.683 (0.360, 1.286)	0.239
		Super-dominant	AA + BB/AB	1.902 (1.000, 3.673)	0.052^#^	1.735 (0.894, 3.408)	0.105
		Homozygote	AA/BB	1.235 (0.412, 3.948)	0.709	1.132 (0.367, 3.706)	0.831
		The allele	A/B	0.830 (0.508, 1.353)	0.456	0.818 (0.474, 1.408)	0.468
5	*FOXO3*	rs10457180					
		Recessive	BB/AA + AB	1.758 (0.621, 5.395)	0.298	1.617 (0.564, 5.023)	0.381
		Dominant	AA/BB + AB	0.710 (0.382, 1.311)	0.274	0.762 (0.403, 1.439)	0.403
		Super-dominant	AA + BB/AB	1.810 (0.950, 3.498)	0.074^#^	1.636 (0.839, 3.223)	0.150
		Homozygote	AA/BB	1.433 (0.491, 4.515)	0.518	1.337 (0.448, 4.297)	0.609
		The allele	A/B	0.912 (0.560, 1.483)	0.710	0.918 (0.535, 1.575)	0.757
6	*GAPDH*	rs6489721					
		Recessive	BB/AA + AB	1.000 (0.441, 2.268)	1.000	1.009 (0.439, 2.324)	0.984
		Dominant	AA/BB + AB	0.727 (0.381, 1.377)	0.329	0.732 (0.366, 1.453)	0.373
		Super-dominant	AA + BB/AB	1.336 (0.727, 2.469)	0.352	1.314 (0.696, 2.490)	0.400
		Homozygote	AA/BB	0.813 (0.327, 2.013)	0.652	0.946 (0.356, 2.551)	0.911
		The allele	A/B	0.861 (0.555, 1.334)	0.503	0.801 (0.477, 1.341)	0.399
7	*GJB2*	rs3751385					
		Recessive	BB/AA + AB	**2.436 (1.232, 4.951)**	**0.012**	**2.553 (1.260, 5.348)**	**0.011**
		Dominant	AA/BB + AB	1.181 (0.615, 2.280)	0.618	1.333 (0.681, 2.629)	0.402
		Super-dominant	AA + BB/AB	1.851 (0.987, 3.516)	0.057^#^	1.715 (0.898, 3.309)	0.104
		Homozygote	AA/BB	2.108 (0.957, 4.748)	0.067^#^	**2.349 (1.030, 5.532)**	**0.046**
		The allele	A/B	**1.546 (1.004, 2.389)**	**0.049**	**1.798 (1.111, 2.933)**	**0.018**
8	*HDAC2*	rs10499080					
		Recessive	BB/AA + AB	0.850 (0.381, 1.878)	0.687	0.775 (0.339, 1.753)	0.540
		Dominant	AA/BB + AB	0.544 (0.269, 1.080)	0.085^#^	0.550 (0.261, 1.135)	0.109
		Super-dominant	AA + BB/AB	1.476 (0.801, 2.739)	0.213	1.371 (0.719, 2.626)	0.338
		Homozygote	AA/BB	0.563 (0.219, 1.420)	0.225	0.589 (0.218, 1.571)	0.290
		The allele	A/B	0.746 (0.483, 1.151)	0.186	0.623 (0.364, 1.056)	0.081^#^
9	*HOTAIR*	rs874945					
		Recessive	BB/AA + AB	-	-	-	-
		Dominant	AA/BB + AB	1.114 (0.585, 2.127)	0.743	1.108 (0.550, 2.144)	0.817
		Super-dominant	AA + BB/AB	0.672 (0.343, 1.301)	0.240	0.731 (0.360, 1.467)	0.381
		Homozygote	BB/AA	-	-	-	-
		The allele	A/B	0.886 (0.506, 1.546)	0.671	0.896 (0.475, 1.684)	0.733
10	*hOGG1*	rs1052133					
		Recessive	BB/AA + AB	0.546 (0.281, 1.048)	0.071^#^	0.580 (0.293, 1.135)	0.114
		Dominant	AA/BB + AB	0.721 (0.320, 1.593)	0.421	0.674 (0.288, 1.539)	0.352
		Super-dominant	AA + BB/AB	0.713 (0.386, 1.311)	0.278	0.780 (0.415, 1.460)	0.438
		Homozygote	AA/BB	0.510 (0.204, 1.248)	0.143	0.494 (0.188, 1.261)	0.144
		The allele	A/B	1.449 (0.937, 2.248)	0.096^#^	1.610 (0.960, 2.725)	0.073^#^
11	*PTPRN2*	rs10081191					
		Recessive	BB/AA + AB	1.897 (0.626, 6.418)	0.270	2.083 (0.653, 7.428)	0.228
		Dominant	AA/BB + AB	0.679 (0.367, 1.249)	0.215	0.659 (0.346, 1.242)	0.199
		Super-dominant	AA + BB/AB	1.810 (0.976, 3.389)	0.061^#^	**1.961 (1.020, 3.831)**	**0.046**
		Homozygote	AA/BB	1.473 (0.466, 5.155)	0.520	1.480 (0.435, 5.577)	0.540
		The allele	A/B	0.892 (0.557, 1.426)	0.632	0.839 (0.486, 1.442)	0.525
12	*SIK3*	rs6589574					
		Recessive	BB/AA + AB	0.468 (0.140, 1.383)	0.184	0.482 (0.141, 1.480)	0.214
		Dominant	BB + AB/AA	**2.186 (0.244, 0.847)**	**0.014**	**2.355 (1.247, 4.523)**	**0.009**
		Super-dominant	AA + BB/AB	1.725 (0.930, 3.229)	0.085^#^	1.892 (0.999, 3.632)	0.052^#^
		Homozygote	AA/BB	3.000 (0.972, 10.398)	0.064^#^	2.938 (0.899, 10.656)	0.082^#^
		The allele	A/B	**1.830 (1.141, 2.959)**	**0.013**	**2.242 (1.292, 3.948)**	**0.005**
13	*STAT3*	rs1053023					
		Recessive	BB/AA + AB	2.637 (0.928, 8.627)	0.082^#^	**5.207 (1.103, 32.315)**	**0.049**
		Dominant	AA/BB + AB	1.052 (0.562, 1.974)	0.873	1.101 (0.566, 2.144)	0.776
		Super-dominant	AA + BB/AB	0.679 (0.367, 1.249)	0.215	0.704 (0.369, 1.334)	0.283
		Homozygote	AA/BB	0.430 (0.125, 1.307)	0.152	0.596 (0.159, 2.053)	0.420
		The allele	A/B	0.855 (0.545, 1.338)	0.493	0.849 (0.485, 1.482)	0.566
14	*STAT3*	rs1053005					
		Recessive	BB/AA + AB	**3.338 (1.106, 12.367)**	**0.045**	**6.038 (1.223, 40.273)**	**0.039**
		Dominant	AA/BB + AB	1.000 (0.534, 1.871)	1.000	1.039 (0.536, 2.015)	0.908
		Super-dominant	AA + BB/AB	0.679 (0.367, 1.249)	0.215	0.704 (0.369, 1.334)	0.283
		Homozygote	BB/AA	3.000 (0.933, 11.637)	0.081^#^	2.278 (0.633, 9.517)	0.224
		The allele	A/B	0.810 (0.516, 1.270)	0.359	0.784 (0.446, 1.371)	0.394
15	Table 2	rs521845					
		Recessive	BB/AA + AB	0.753 (0.314, 1.768)	0.516	0.786 (0.320, 1.902)	0.592
		Dominant	AA/BB + AB	1.512 (0.805, 2.865)	0.200	1.550 (0.811, 2.987)	0.186
		Super-dominant	AA + BB/AB	0.586 (0.315, 1.080)	0.088^#^	0.584 (0.308, 1.097)	0.096^#^
		Homozygote	BB/AA	1.019 (0.394, 2.594)	0.969	1.190 (0.437, 3.246)	0.732
		The allele	A/B	1.135 (0.730, 1.768)	0.574	1.218 (0.725, 2.054)	0.457

Bold values indicate statistical significance

* OR and 95% CI were calculated by logistic regression analyses (unadjusted)

† OR_adj_ and 95% CI were calculated by logistic regression analyses and adjusting for age, year of noise exposure, smoking and drinking status

# 0.05 ≤ *p* < 0.10

AA, wild genotype; AB, heterozygous mutation genotype; BB, homozygous mutant genotype

### Six candidate SNPs were verified in the replication stage

In this stage, 153 NIHL and 252 controls were enrolled. As shown in Supplementary Table 4, the mean age in NIHL group was significantly higher than in control group (*p* < 0.05). No statistical significance between the cases and controls in terms of sex, ethnicity, CNE and years of noise exposure were observed (*p* > 0.05). The use rate of PPE and drinking rate in the NIHL group were slightly lower than those in the control group (*p* < 0.05). The BHFTA in NIHL group was 55.86 ± 10.72 dB, and was significantly higher than the control group (24.92 ± 8.17 dB; *p* < 0.05).

We further verified the eight candidate SNPs screened from the first stage. Five SNPs in four genes (*CDH23* rs2394795, *FAS* rs1468063 and rs2862833, *GJB2* rs3751385, *SIK3* rs6589574) showed significantly different between two groups under at least one genetic model (*p* < 0.05; Supplementary Table 5). Finally, the variant alleles of *CDH23* rs2394795, *FAS* rs1468063 and rs2862833, *GJB2* rs3751385, *PTPRN2* rs10081191 and *SIK3* rs6589574 showed significantly association with NIHL risk in logistic regression model after adjusting for multiple variables (Table 2).

**Table 2:** ORs and 95% CI of NIHL-associated SNPs in the replication stage

Code	Gene	SNPs	Genotype	OR (95% CI)^*^	*p*	OR_adj_ (95% CI)^†^	*p*
1	*CDH23*	rs2394795					
		Recessive	BB/AA + AB	**2.261 (1.361, 3.872)**	**0.002**	**2.358 (1.336, 4.313)**	**0.004**
		Dominant	AA/BB + AB	1.562 (0.954, 2.550)	0.075^#^	1.527 (0.869, 2.674)	0.139
		Super-dominant	AA + BB/AB	0.777 (0.516, 1.167)	0.226	0.739 (0.467, 1.163)	0.194
		Homozygote	BB/AA	**2.704 (1.437, 5.181)**	**0.002**	**3.024 (1.438, 6.549)**	**0.004**
		The allele	B/A	**1.522 (1.144, 2.027)**	**0.004**	**1.505 (1.099, 2.065)**	**0.011**
2	*FAS*	rs1468063					
		Recessive	AA + AB/BB	**1.861 (1.124, 3.085)**	**0.016**	**1.950 (1.113, 3.423)**	**0.019**
		Dominant	AA/BB + AB	0.813 (0.540, 1.228)	0.324	0.756 (0.477, 1.197)	0.233
		Super-dominant	AA + BB/AB	**1.804 (1.221, 2.816)**	**0.004**	**2.077 (1.302, 3.356)**	**0.002**
		Homozygote	AA/ BB	1.453 (0.837, 2.527)	0.184	1.427 (0.772, 2.639)	0.255
		The allele	A/B	1.106 (0.827, 1.476)	0.496	1.084 (0.785, 1.495)	0.623
3	*FAS*	rs2862833					
		Recessive	AA + AB/BB	1.200 (0.788, 1.821)	0.394	1.349 (0.844, 2.156)	0.211
		Dominant	BB + AB/AA	**1.639 (1.013, 2.650)**	**0.043**	**1.722 (1.008, 2.942)**	**0.046**
		Super-dominant	AB/AA + BB	**1.693 (1.123, 2.571)**	**0.013**	**2.006 (1.256, 3.248)**	**0.004**
		Homozygote	BB/AA	1.306 (0.763, 2.238)	0.330	1.205 (0.661, 2.193)	0.540
		The allele	B/A	1.093 (0.820, 1.455)	0.543	1.054 (0.766, 1.448)	0.746
4	*GJB2*	rs3751385					
		Recessive	AA + AB/BB	**2.659 (1.686, 4.218)**	**< 0.001**	**3.061 (1.823, 5.175)**	**< 0.001**
		Dominant	AA/BB + AB	**1.712 (1.097, 2.709)**	**0.020**	**2.119 (1.289, 3.551)**	**0.034**
		Super-dominant	AB/AA + BB	1.381 (0.919, 2.084)	0.122	1.270 (0.807, 2.007)	0.303
		Homozygote	AA/ BB	**2.981 (1.736, 5.182)**	**< 0.001**	**3.731 (2.004, 7.150)**	**< 0.001**
		The allele	A/B	**1.836 (1.379, 2.450)**	**< 0.001**	**2.106 (1.531, 2.909)**	**< 0.001**
5	*PTPRN2*	rs10081191					
		Recessive	AA + AB/BB	1.109 (0.560, 2.146)	0.760	1.610 (0.760, 3.352)	0.205
		Dominant	AA/BB + AB	1.370 (0.916, 2.052)	0.126	1.443 (0.921, 2.269)	0.110
		Super-dominant	AB/AA + BB	1.437 (0.955, 2.173)	0.084^#^	**1.754 (1.105, 2.815)**	**0.018**
		Homozygote	AA/ BB	0.954 (0.459, 1.857)	0.933	1.181 (0.530, 2.588)	0.679
		The allele	A/B	0.850 (0.623, 1.156)	0.304	0.894 (0.634, 1.255)	0.518
6	*SIK3*	rs6589574					
		Recessive	AA + AB/BB	0.804 (0.408, 1.526)	0.515	0.724 (0.345, 1.462)	0.378
		Dominant	AA/BB + AB	**1.550 (1.035, 2.325)**	**0.034**	**1.743 (1.113, 2.749)**	**0.016**
		Super-dominant	AB/AA + BB	1.437 (0.955, 2.173)	0.084^#^	1.542 (0.980, 2.442)	0.063^#^
		Homozygote	AA/ BB	0.654 (0.323, 1.280)	0.224	0.599 (0.272, 1.267)	0.189
		The allele	A/B	1.351 (0.994, 1.844)	0.056^#^	**1.472 (1.050, 2.077)**	**0.026**
7	*STAT3*	rs1053023					
		Recessive	AA + AB/BB	0.632 (0.325, 1.172)	0.157	0.823 (0.408, 1.633)	0.600
		Dominant	AA/BB + AB	1.168 (0.778, 1.761)	0.455	1.440 (0.914, 2.289)	0.119
		Super-dominant	AA + BB/AB	1.418 (0.947, 2.127)	0.090^#^	1.539 (0.985, 2.418)	0.059^#^
		Homozygote	AA/ BB	0.731 (0.363, 1.416)	0.364	1.030 (0.478, 2.172)	0.938
		The allele	A/B	0.976 (0.724, 1.313)	0.874	1.159 (0.835, 1.610)	0.377
8	*STAT3*	rs1053005					
		Recessive	AA + AB/BB	0.632 (0.325, 1.172)	0.157	0.823 (0.408, 1.633)	0.600
		Dominant	AA/BB + AB	1.168 (0.778, 1.761)	0.455	1.440 (0.914, 2.289)	0.119
		Super-dominant	AA + BB/AB	1.418 (0.947, 2.127)	0.090^#^	1.539 (0.985, 2.418)	0.059^#^
		Homozygote	AA/ BB	0.731 (0.363, 1.416)	0.364	1.030 (0.478, 2.172)	0.938
		The allele	A/B	0.976 (0.724, 1.313)	0.874	1.159 (0.835, 1.610)	0.377

Bold values indicate statistical significance

* OR and 95% CI were calculated by logistic regression analyses (unadjusted)

† OR_adj_ and 95% CI were calculated by logistic regression analyses and adjusting for age, year of noise exposure, smoking and drinking status

# 0.05 ≤ *p* < 0.10

AA, wild genotype; AB, heterozygous mutation genotype; BB, homozygous mutant genotype

### Genotyping and calculation of GRS

The remaining six SNPs mentioned above were used to build the GRS model. Different distributions of GRS values were presented between NIHL cases and the controls (Table 3). The median (P_25_, P_75_) of GRS in the NIHL group was 7.0 (5.0, 8.0), and was significantly higher than the control group [5.0 (4.0, 7.0)] (*p* < 0.001). The proportion of NIHL subjects was gradually higher than controls as the increase of GRS value. When the individuals were divided based on GRS values, the lowest levels of GRS (≤ 5) as the reference group, the positive trend and significant association of the GRS values with NIHL risk was observed (*p* < 0.05). The individuals were further divided into high and low GRS group according to a GRS value of 6, then the subjects with GRS ≥ 6 had increased risk for NIHL, with an OR of 2.734 (95%CI = 1.801, 4.195) compared to the subjects with GRS < 6.

**Table 3:** Associations between levels of GRS and risk for NIHL.

GRS	total (*n* = 405)	NIHL (*n* = 153)	Control (*n* = 252)	*p*	OR (95% CI)
Median (P_25_, P_75_)	6.0 (4.0, 7.0)	7.0 (5.0, 8.0)	5.0 (4.0, 7.0)	< 0.001	
GRS subgroup [n (%)]					
≤ 5	188 (46.4)	48 (31.4)	140 (55.6)	-	1.000
6	35 (14.1)	22 (14.4)	35 (13.9)	0.058^#^	1.833 (0.973, 3.417)
7	63 (15.6)	31 (20.3)	32 (12.7)	**0.001**	**2.826 (1.563, 5.131)**
8	52 (12.8)	22 (14.4)	30 (11.9)	**0.020**	**2.139 (1.1211, 4.054)**
9	27 (6.7)	17 (11.1)	10 (4.0)	**< 0.001**	**4.958 (2.160, 11.948)**
≥ 10	18 (4.4)	13 (8.5)	5 (2.0)	**< 0.001**	**7.583 (2.708, 24.661)**
*p*_trend_				0.043	
< 6	188 (46.4)	48 (31.4)	140 (55.6)		1.000
≥ 6	217 (53.6)	105 (68.6)	112 (44.4)	**< 0.001**	**2.734 (1.801, 4.195)**

Bold values indicate statistical significance

# 0.05 ≤ *p* < 0.10

### CART analysis

CART analysis was performed through incorporating both the genetic and other variables. Compared to individuals carried *GJB2* rs3751385 (AA/BB) variant genotypes, individuals carried *GJB2* rs3751385 (BB), *FAS* rs1468063 (AA/BB) and *PTPRN2* rs10081191(AA) variant genotypes had higher risk of NIHL (OR = 4.250; 95% CI = 1.984, 9.539; *p* < 0.001), and individuals carried *GJB2* rs3751385 (BB) and *FAS* rs1468063 (AA/BB) variant genotypes had the highest risk of NIHL (OR = 12.084; 95% CI = 3.923, 46.526; *p* < 0.001; Table 4). Table 5 depicted the resultant tree structure generated. There was an initial split on *GJB2* rs3751385, suggesting that *GJB2* rs3751385 is the most important risk factor for NIHL. Further analysis of the CART structure found distinct patterns for *GJB2* rs3751385 (AA/BB) and *GJB2* rs3751385 (BB). Compared to the reference group (individuals with age < 45 years and carried *GJB2* rs3751385 (AA/BB)), individuals carried *GJB2* rs3751385 (BB) and *PTPRN2* rs10081191 (AA) variant genotypes had the higher risk of NIHL (OR = 7.585; 95%CI = 3.654, 16.443; *p* < 0.001; terminal node 8), and individuals carried *GJB2* rs3751385 (BB) and *PTPRN2* rs10081191 (BB/AB) had a 2.483-fold increased risk of NIHL (95% CI = 1.239, 4.998; *p* < 0.05; terminal node 7). For age ≥ 45, individuals carried *GJB2* rs3751385 (AA/AB) and *FAS* rs1468063 (BB) in variant genotypes had a 3.012-fold increased risk of NIHL (95%CI = 1.389, 6.567; *p* < 0.05; terminal node 6), individuals with CNE < 95 (dB(A)·years), without PPE and carried *GJB2* rs3751385 (AA/AB) and *FAS* rs1468063 (AA/AB) variant genotypes had a 10.038 -fold increased risk of NIHL (95%CI = 2.770, 47.792; *p* < 0.05; terminal node 5). For non-drinkers, individuals with age ≥ 45 years and CNE < 95 (dB(A)·years) carried *GJB2* rs3751385 (AA/AB) and *FAS* rs1468063 (AA/AB) variant genotypes had a 2.677-fold increased risk of NIHL (95% CI = 1.103, 6.447; *p* < 0.05; terminal node 3).

**Table 4:** Risk estimate of gene-gene interaction in CART model

Terminal Node	Combinations of SNPs			n (Control/NIHL)	OR (95% CI)	*p* ^*^
	*GJB2* rs3751385	*FAS* rs1468063	*PTPRN2* rs10081191			
1	AA/AB			206/96	1.000	
2	BB	AA/AB	BB/AB	28/14	1.164 (0.527, 2.474)	0.699
3	BB	AA/AB	AA	14/27	**4.250 (1.984, 9.539)**	**< 0.001**
4	BB	BB		4/16	**12.084 (3.923, 46.526)**	**< 0.001**

Bold values indicate statistical significance

* Adjusted for age, sex, nationality, year of noise exposure, CNE, use of PPE, drinking and smoking status

AA, wild genotype; AB, heterozygous mutation genotype; BB, homozygous mutant genotype

**Table 5:** Risk estimate of gene-environment interaction in CART model

Terminal Node	Combinations of environment				Combinations of SNPs			n (Control/NIHL)	OR (95% CI)	*p*
	PPE	CNE	Drinking	Age	*GJB2* rs3751385	*FAS* rs1468063	*PTPRN2* rs10081191			
1				< 45	AA/AB			87/26	1.000	-
2		< 95	Yes	≥ 45	AA/AB	AA/AB		18/9	1.673 (0.651, 4.102)	0.269
3		< 95	No	≥ 45	AA/AB	AA/AB		15/12	**2.677 (1.103, 6.447)**	**0.028**
4	Yes	≥ 95		≥ 45	AA/AB	AA/AB		63/22	1.168 (0.604, 2.247)	0.641
5	No	≥ 95		≥ 45	AA/AB	AA/AB		3/9	**10.038 (2.770, 47.792)**	**0.001**
6				≥ 45	AA/AB	BB		20/18	**3.012 (1.389, 6.567)**	**0.005**
7					BB		BB/AB	31/23	**2.483 (1.239, 4.998)**	**0.010**
8					BB		AA	15/34	**7.585 (3.654, 16.443)**	**< 0.001**

Bold values indicate statistical significance

AA, wild genotype; AB, heterozygous mutation genotype; BB, homozygous mutant genotype

## Discussion

In present study, we have applied a multigenic approach to systematically explore the relationships between polymorphisms in susceptibility genes and NIHL risk. We screened and validated 6 NIHL-associated SNPs in five genes from 60 candidate-SNPs in a two-stage case-control study. The most important discovery in this study is that joint analyses of multiple SNPs in multiple susceptibility genes may find otherwise undetectable correlations between a single SNP and NIHL risk. Moreover, we contructed a genetic risk predictive model according to these SNPs and found a dose-response relationship between GRS values and NIHL risk. Our findings indicate that a more comprehensive multigenic approach combining multiple polymorphisms provides more accurate delineation of risk groups and may suggest the future direction of correlation studies.

Many studies have reported the relationships between genetic polymorphisms and susceptibility to complex diseases like NIHL. With the progress of science and technology, more and more genomic methods were applied to explore the genetic susceptibility of NIHL [16], such as MALDI-TOF-MS [17], TaqMan probe method [18], Sanger sequencing [19], SNaPshot sequencing [20], exome-wide association study (EWAS) [21] and genome wide association study (GWAS) [22, 23]. In this study, SNaPshot sequencing was used in the screening stage and Taqman-MGB probe method was conducted in the replication stage for genotyping. Most studies have conducted a candidate-gene approach to explore only one or several selected genes at a time. Nevertheless, a large number of positive candidate genes have not been replicated in different populations. Replication in independent sample sets plays a vital role in confirming susceptibility genes for complex diseases. It is now generally believed that replication of findings in several independent sample sets is more important than to obtain highly significant *p* values [24]. Therefore, only findings that caused significant associations in both sample sets were considered meaningfully. Applying this replication criterion may acquire more reliable association results and avoid the problem of multiple testing. In our study, six SNPs in five genes, including *SIK3* rs6589574, *FAS* rs1468063 and rs2862833, *GJB2* rs3751385, *PTPRN2* rs10081191 and *CDH23* rs2394795, showed significant (*p* < 0.05) association with NIHL risk.

NIHL is a polygenic disease involving variants of multiple SNPs in multiple genes, thus the impact of a single polymorphism locus is weak. GRS was used to evaluate the cumulative effect of the genetic markers mentioned above on individual genetic predisposition to NIHL, similar to that conducted on other specific complex diseases, such as cancer and diabetes [25, 26]. Our data indicated that NIHL cases have a higher genetic susceptibility than controls, and these SNPs have a cumulative effect on NIHL genetic risk. In addition, a positive dose–response relationship between GRS levels and NIHL risk was observed. These findings extend our understanding of the relationship between multiple genes and the susceptibility of NIHL. Using these genetic biomarkers, we could screen individuals susceptible to NIHL, and distinguish higher sensitivity to NIHL from the noise-exposed workers. It is relatively difficult to avoid noise exposure in most work environments. Thus, effective and efficient preventive measures for high-risk populations are very important. In this case, the susceptible individuals should be screened and identified, and appropriate measures can be taken to reduce noise exposure and strengthen protection (wearing earplugs or earmuffs) in the noise environment, so as to effectively reduce the risk of NIHL.

Since environment factors such as sex, age, ethnicity and lifestyle may interact with genetic factors to promote the occurrence of NIHL, the impact of each individual SNP is unlikely to be substantial [27, 28]. Therefore, CART was conducted to analysis higher-order interactions among gene-gene, gene-environment. Compared to individuals carried *GJB2* rs3751385 (AA/BB) variant genotypes, individuals carried *GJB2* rs3751385 (BB), *FAS* rs1468063 (AA/BB) and *PTPRN2* rs10081191 (AA) variant genotypes had a 4.250-fold increased NIHL risk (95% CI = 1.984, 9.539), and individuals carried *GJB2* rs3751385 (BB) and *FAS* rs1468063 (AA/BB) variant genotypes had a 12.084-fold increased NIHL risk (95% CI = 3.923, 46.526). We also observed a significant multiplicative interaction between age and three SNPs. All genetic effects were apparent only in “age ≥ 45” (subjects aged ≥ 45 years ) and not in “age < 45”. More interestingly, subgroups of individuals with higher NIHL risks were found according to simple combinations of age and genotypes. Our data presented an interaction between rs3751385, rs1468063, rs10081191, age, CNE, PPE and drinking status. Aging can aggravate the apoptosis of hair cells in cochlear, especially for the elderly exposed to noise, which may increase the prevalence of NIHL. It was reported that noise exposed workers aged 45–59 have a higher risk of NIHL than those aged 30–44 [29]. In addition, an obvious dose-response relationship between CNE and hearing loss has been confirmed [30]. It has been confirmed that drinking can increase the risk of NIHL, which may be related to vasospasm and contraction of inner ear terminals caused by excessive drinking, as well as ischemia and hypoxia of cochlear cells [31, 32]. It was well known that the easiest method to prevent NIHL is to wear earmuffs or earplugs. A study showed that in the logistic regression analysis, there was a significant difference in the rates of hearing loss between wearing earplugs sometimes (OR = 1.48, 95% CI = 1.07, 2.05) and never wearing earplugs (OR = 1.53, 95% CI = 1.12, 2.10) [33]. CART algorithm may rapidly identify the potential genetic and environmental interactions when dealing with numerous variables in complex diseases. However, the CART analysis is a postdata-mining tool and the results should be interpreted more cautious.

Briefly, six SNPs in five genes showed significant association with risk of NIHL in our study. Interestingly, all of them were non-coding SNPs, which may cause single base changes, resulting in 3’-UTR or intron variants, ultimately altering gene expression levels rather than proteins. The current studies on disease-related SNPs mainly focus on the regulatory regions or coding regions of the genome. However, in the human genome, SNPs in non-coding regions are more than in coding regions. The 3’ untranslated region (3’UTR) is crucial for the genes transcriptional regulation [34]. Gene variation in 3’UTR does not directly change the coding sequence of the gene, but can cause the change of gene function. MiRNAs are required to regulate gene expression by binding to the 3’UTR of target mRNAs. SNPs in 3’UTR can affect the recognition of miRNAs and target genes and the expression level of mature miRNAs, so as to enhance the expression of target gene by weakening the translation inhibitory effect of miRNA on target gene, or inhibit the expression of target gene by forming new miRNA binding sites. A recent study based on a large sample noise exposure cohort has reported that a variation in non-coding regions of gene adaptor-associated kinase 1 (*AAK1*) associated with the susceptibility of NIHL. Individuals with *T/T* genotype of *AAK1* rs1396793 had stronger resistance to NIHL than those with *G/T* or *G/G* genotype, which may related to the upregulation of expression of AAK1. Compared with the *AAK1*^*G/G*^ and *AAK1*^*T/G*^ mice, the *AAK1*^*T/T*^ mice also showed significantly less auditory brainstem response (ABR) threshold elevation and wave I amplitude decrement at higher frequencies after noise exposure, which may provide a new target for the prevention and treatment of NIHL [21]. Our data suggest an interaction between *GJB2*, *FAS* and *PTPRN2*. We speculate that one or more transcriptional regulators with similar functions and structures may simultaneously regulate these four genes expression levels, which together act to enhance the risk of NIHL. Therefore, these findings suggested that the detection of non-coding SNPs is also of great significance for the study of disease-related SNPs.

GJB2 protein encoded by *GJB2* (*Cx26*) has about 220 mutation SNPs associated with hearing loss. The mutations of *GJB2* were closely related to late-onset progressive hearing loss, especially among East Asia populations [35]. *GJB2*, as a potassium circulation channel gene, is closely related to NIHL and has been validated in various populations [3, 36]. *GJB2* knock-in mice were more prone to NIHL, which may be due to the reduction of cochlear amplifier caused by lowered endocochlear potential, or the excitotoxicity of inner hair cells induced by potassium accumulation around hair cells [37].

The *FAS* gene belongs to the tumor necrosis factor receptor superfamily, and plays an essential role in the physiological regulation of programmed cell death [38]. *FAS* gene polymorphisms have been mainly studied in association with malignancies and immune system diseases [39, 40]. A previous study confirmed that the genetic polymorphisms in the *FAS* gene are associated with NIHL risk [41]. Noise can induce large amounts of reactive oxygen species (ROS) in cochlear tissue and blood, leading to oxidative stress, lipid peroxidation, and DNA damage, and further activating the *FAS* gene. The mutation of 3’-UTR of *FAS* resulted in the failure to suppress the high expression of FAS under noise stress, which eventually led to the apoptosis of hair cells.

*PTPRN2* is a member of the Protein Tyrosine Phosphatases family (PTPs). PTPRN2 is an autoantigen in insulin-dependent diabetes and has been mainly studies in relation to metabolic diseases such as obesity, diabetes, and cancer [42, 43]. PTPRN2 has been shown to be linked to severe bilateral hearing loss [44, 45]. A Chinese population study showed an increased prevalence of NIHL in individuals with *PTPRN2*-rs10081191 allele A [23]. Recently, an increasing number of PTPs is identified as clinically relevant targets [45]. Thus larger population samples and more in-depth experimental studies are needed to confirm the mechanism of *PTPRN2* in hearing loss.

*CDH23* encodes a member of the cadherin super-family and has been validated associations with NIHL risk [1, 3]. The CDH23 protein is strongly associated with the structure and function of the inner ear and is essential for cellular adhesion. The mutation of *CDH23* changes the cellular adhesion in inner hair-cells, reduces the stability of cadherin and increases potassium (K^+^) influx and frequent depolarization, which may cause the opening of the calcium ion channel on the basolateral membrane and the entery of excessive calcium ions, resulting in hair-cells death and hearing loss [46, 47].

*SIK3* is a subfamily of serine/threonine protein kinase and is widely involved in glucose, cholesterol and lipid metabolism. *SIK3* plays an important role in the early development of hair cells or sterecilia, and plays a maintenance function of non-neural cells throughout development and adulthood of the spiral ganglion [26, 41]. A meta-analysis of the whole genome related to hearing function from G-EAR Association and Twins UK found that rs681524 locus of *SIK3* gene is closely related to hearing function of European population [48]. Another case-control study suggested that 3 SNPs (rs493134, rs6589574, and rs7121898) of *SIK3* may be an important part of NIHL susceptibility [49].

This study had several advantages that could improve the robustness of our findings. First, two independent sample sets were used to confirm the true association between NIHL susceptibility and genetic polymorphisms, which may improve the reproducibility and power of the study. Second, GRS was employed as an risk prediction model to screen subjects sensitive to NIHL, so as to decrease the risk of NIHL. In addition, a multigenic approach such as CART model was used to estimate the gene-gene, gene-enviromnent interaction to more comprehensively understand the risk of NIHL. However, the shortcoming of this study is that the sample size remains limited. Further studies are warranted to confirm our findings.

### Electronic supplementary material

Below is the link to the electronic supplementary material.

Supplementary Table 1: General information of 60 SNPs in NIHL candidate susceptible genes

Supplementary Table 2: General demographic characteristics of subjects in screening stage

Supplementary Table 3: Distribution of genotypes of the selected SNPs and the results of Hardy-Weinberg test

Supplementary Table 4: General demographic characteristics of subjects in replication stage

Supplementary Table 5: Distribution of genotypes of 8 NIHL-associated SNPs and the results of Hardy-Weinberg test

## Data Availability

The data reported in this paper have been deposited in the OMIX, China National Center for Bioinformation / Beijing Institute of Genomics, Chinese Academy of Sciences (https://ngdc.cncb.ac.cn/omix), and the OMIX ID (shared link) of the data of screening stage and replication stage is OMIX005285 (https://ngdc.cncb.ac.cn/omix/preview/QtCW2nhS) and OMIX005284 (https://ngdc.cncb.ac.cn/omix/preview/GvGTuAln), respectively.
