# Supplementary material for: Interactions of genetic variations in FAS, GJB2 and PTPRN2 are associated with noise-induced hearing loss: a case-control study in China
Source: BMC Med Genomics. 2024 Jan 11;17:18. doi: 10.1186/s12920-023-01790-7 (PMC10785407; doi:10.1186/s12920-023-01790-7)
Supplement: Supplementary file 1 — Supplementary Table 1: General information of 60 SNPs in NIHL candidate susceptible genes [file 12920_2023_1790_MOESM1_ESM.docx]

**Supplementary Table 1.** General information of 60 SNPs in NIHL candidate susceptible genes.

| **Code** | **Categories** | **Gene** | **Chr.** | **SNPs ID**  **(A/B)** | **Primer Sequences (5’→3’)** |
| --- | --- | --- | --- | --- | --- |
| 1 | Oxidative Stress Genes | *CAT* | 11 | rs7943316  (A/T) | F: AGCCAATCAGAAGGCAGTCC  R: GCTTTCTAAACGGACCTTCG  S: TTTTTTTTTTTTTGCTGAGCCTGAAGTCGCCACGG |
| 2 |  | *CAT* | 11 | rs769214  (A/G) | F: CCTGAGGAGGTGTAGAAATC  R: GATGGGTGTTGATTTCCTCC  S: TCAAAATTCCTGCTTACCTGGG |
| 3 |  | *CAT* | 11 | rs769217  (C/T) | F: TTGCCTATCCTGACACTCAC  R: AAAAAGCGCCCAGTTAGCAG  S: TTTTTTTTTTTTTTTTTTTTGAGTGGCCAACTACCAG CGTGA |
| 4 |  | *PON3* | 7 | rs11767787  (G/A) | F: TTAAGGGCCCAGATTTTGGAG  R: CCCTCTTTCTTCTAGGTCTTG  S: AAATTGGGGTAAGGCTACCCAC |
| 5 |  | *PON3* | 7 | rs13226149  (G/A) | F: TGACCTCACTTGGAAGAGGAG  R: TCAGGTAAGTAGGAGGCGTGTG  S: TTTTTTTTTTATGCCACTCACCTAAACGCCAG |
| 6 |  | *PON3* | 7 | rs17882539  (C/T) | F: TTAAGGGCCCAGATTTTGGAG  R: CCCTCTTTCTTCTAGGTCTTG  S: CCTCCCCCTCCAACCTGGTGTT |
| 7 |  | *NFE2L2* | 2 | rs6726395  (G/A) | F: GGTTGCAAACATCTTCTGCTC  R: GAGAGATACTTTTCACGTGCC  S: TTTTTTTTTTAATTATTCCATCCTACCCAAGC |
| 8 |  | *NFE2L2* | 2 | rs77684420  (T/C) | F: TACCACAACATGCTGTAATCG  R: TGAAGTCTTCTCTGGGCAATG  S: ACATAAAAATCGTTGATTCCAC |
| 9 |  | *NOX3* | 6 | rs12195525  (G/T) | F: CAACAGCCAAAGCCAAATCAG  R: CGCACTGTGCCTGAATTTTAAC  S: TTTTTTTTTTCTTATGAATGAAATAAGGTTTC |
| 10 | Potassium Recycling Channel Genes | *GJB2* | 13 | rs3751385  (C/T) | F: ACGCATTGCCCAGTTGTTAG  R: ACCAATAACCCCTAACAGCC  S: TTTTTTTTTTTTTAATCTTTGTGTTGGGAAATGCT |
| 11 |  | *KCNE1* | 21 | rs1805128  (G/A) | F: GGATTCTTCGGCTTCTTCACC  R: GCCAGATGGTTTTCAACGACA  S: ATTCAACGTCTACATCGAGTCC |
| 12 |  | *KCNE1* | 21 | rs3453  (T/C) | F: CTTTCCTTATTCCCAAGTCCG  R: TCTGGCTTCACATACACAGAG  S: GTTGTCCTAGCTAATGAATGCA |
| 13 |  | *KCNE1* | 21 | rs1805127  (G/A) | F: TGCAGCAGTGGAACCTTAATG  R: CTCGATGTAGACGTTGAATGG  S: TTTTTTTTTTGGCCCGCAGGTCCCCCCGCAGC |
| 14 |  | *KCNQ4* | 1 | rs4660468  (C/T) | F: CTGAGAAAGAAAAGCGAGCC  R: AGCCCTACAAAGACCCTCAC  S: CCTCCTTCCTGGTCTACCTGGC |
| 15 |  | *KCNMA1* | 10 | rs696211  (T/C) | F: GACAGAAGGAGCAGAATCTTG  R: ACCCAGAAACCAAAGCCATTC  S: TTTTTTTTTTGCCATTTTTGGCCTCAAACCCA |
| 16 |  | *KCNMA1* | 10 | rs7910544  (G/C) | F: CCCACTATCACTGTTTTTAGC  R: GACCTAGTAGTTATAGGATAC  S: TTTTTTTTTTTTTTTTTTTTAGGATATAATCAGGTTGA TTTA |
| 17 | Calcium ions Recycling Channel Genes | *PMCA2* | 3 | rs3209637  (C/T) | F: GATGAGGATCAGTATGGCTTG  R: GGGATTACTAGTGCTGTTGTC  S: TTTTTTTTTTCAAACAGAAAGCCATTCTGAAA |
| 18 | Apoptosis Signaling Genes | *CASP3* | 4 | rs1049216  (T/C) | F: ACTTCCAGTCAGGTAGTTGC  R: TTTGAGCCTTTGACCATGCC  S: AAAAAGTTAAACATTGAAGTAA |
| 19 |  | *CASP3* | 4 | rs6948  (A/C) | F: TGTTCTAAAGGTGGTGAGGC  R: GCAACAAGAAATCTCCCGTG  S: TTTTTTTTTTTTTTTTTTCAGCCGGAGGCCAGAGCTG AGC |
| 20 |  | *CASP7* | 10 | rs2227310  (C/G) | F: CCTTTCTTTCCTGTTGAAGGC  R: GTAAATATCCCCTTGGCTGTG  S: TTTTTTTTTTTCCTGGAGGAGCACGGAAAAGA |
| 21 |  | *CASP7* | 10 | rs4353229  (C/T) | F: CCTTTCTTTCCTGTTGAAGGC  R: GTAAATATCCCCTTGGCTGTG  S: ACAGCCATGAGCTTTCTCCAGA |
| 22 |  | *FAS* | 10 | rs1468063  (G/A) | F: TCATGTGCTGTTTGGAAGAGG  R: TTCTTATTTTTCCCCCACCCC  S: TTTTTTTTTTAATCTTAAATCTTAGAAACTTG |
| 23 |  | *FAS* | 10 | rs2862833  (T/C) | F: TCATGTGCTGTTTGGAAGAGG  R: TTCTTATTTTTCCCCCACCCC  S: TTTTTTTTTTTTCTAAGGGATCCAAGAAGCAT |
| 24 | DNA Damage Repair Genes | *hOGG1* | 3 | rs1052133  (C/G) | F: ACACTGTCACTAGTCTCACC  R: TTTGAGGTAGTCACAGGGAG  S: TTTTTTTTTTTTTTTTTTTTTTTTTCAGTGCCGACCTG CGCCAAT |
| 25 |  | *APEX1* | 14 | rs1130409  (G/T) | F: ACGGCATAGGTGAGACCCTA  R: GCTGTTACCAGCACAAACGA  S: TTTTTTTTTTTTTTTTTTTTGGCCTTCCTGATCATGCT CCTC |
| 26 |  | *APEX1* | 14 | rs1760944  (C/A) | F: AGGACACTTAGGTCCCCAAC  R: CAGATAGCACTGGGAAAGAC  S: TTTTTTTTTTTTTACACTGACTTAAGATTCTAACT |
| 27 |  | *XRCC1* | 19 | rs1799782  (C/T) | F: CAGACAAAGATGAGGCAGAGG  R: TCAGACCCAGGAATCTGAGC  S: TTTTTTTTTTTTTTTTTTTTGCCGGGGGCTCTCTTCTT CAGC |
| 28 | Monogenic NIHL Genes | *PCDH15* | 10 | rs7095441  (C/T) | F: TGGCCTGAAGCAAATGGAAG  R: GGTGTATTTTGGAGTTCTGG  S: TATAGTCATAAACTTTTGCTGA |
| 29 |  | *PCDH15* | 7 | rs1104085  (C/T) | F: ACCAACCTGTAACCCAATCC  R: TGAGGTAGGAGAATCCCTTG  S: TTGCACTCCAGCCTGGGCTACA |
| 30 |  | *CDH23* | 10 | rs3752752  (G/A) | F: ACCAGGTTGATGTAGTAGGG  R: CAACACTGGAACTCTCTCTC  S: CCTTCCACGTGGGGTGGTTGTC |
| 31 |  | *CDH23* | 10 | rs2394795  (T/C) | F: ATTAGCAGCCCTAATGGTGTC  R: GACATGAGAGAATAGAGCAGG  S: GTGAATAATCACCAAATTGTAT |
| 32 |  | *VANGL1* | 1 | rs4128133  (T/C) | F: TACAACCACTTGCCGTCATTC  R: GCATCACTAAGTCAAGACACC  S: TTTTTTTTTTTGGTGACAATGACAACACCACA |
| 33 |  | *MYH14* | 19 | rs667907  (T/C) | F: CAGGATTGCCTGATCTGAAC  R: GCATAATTCCTCGTAGCACC  S: TTGATTTAATTCTCATAAGCAA |
| 34 |  | *MYH14* | 19 | rs588035  (C/G) | F: TACAGCAACAGGTCAACAGG  R: TGTGTACAGGCAGCAAGAAG  S: TTTTTTTTTTTTTTTTTTTTTTTTCATGGCAGGTTAAT GGGTTGG |
| 35 |  | *WHRN* | 9 | rs12339210  (G/C) | F: ACTAGAATGGTGAGCTTGTGC  R: TGCTCACTTTGCTTTCTCGTC  S: TTTTTTTTTTGGCCTTACCACGGACACATCTG |
| 36 | Transcription Factors | *POU4F3* | 5 | rs891969  (G/A) | F: GGTTATCGGGAACTCCAAGG  R: GGCTCCTGCTCTGAAGTCG  S: TTTTTTTTTTTGGGAAAATATTGCAGAAGGGC |
| 37 |  | *FOXO3* | 6 | rs2802292  (T/G) | F: AAACTGAGGCTAACAGCTGG  R: ATGCTCCTCAACGAAACCAC  S: TTTTTTTTTTTTTTCACAAGAGCTCAGGGCTGGGA |
| 38 |  | *FOXO3* | 6 | rs10457180  (A/G) | F: TTGCTTCACCTTGTCCTTGC  R: GGAACAGTCCTTCCATCTTG  S: TTTTTTTTTTTTTTTTTTTTTTTGTTCAGCTGTGTTTG CCCCTCA |
| 39 |  | *GRHL2* | 8 | rs666026  (T/G) | F: AGCCTAGACCAATAGAAAGCC  R: ACACCTATTCTTCTGCCAGAG  S: GAGTGTTTTAATGGGAAAGAAG |
| 40 |  | *HOTAIR* | 12 | rs874945  (G/A) | F: CCAGTTTCTTGGCTCCTATG  R: ATCTGTCCAGTCGCTCGTC  S: TTTTTTTTTTTTTTCAGACTCCAGCCGCTCTTGGA |
| 41 | Inflammatory Factor Genes | *CARD8* | 19 | rs2043211  (A/T) | F: GACACCTCCATGGAAGAAAAC  R: CCCTGTGTTTCTGAGACCCTTTG  S: TTTTTTTTTTTGACACTCAGGAACAGCACGGA |
| 42 |  | *HDAC2* | 6 | rs10499080  (C/T) | F: CAAACCAAACATTTGACCTCCC  R: GTTCAGCCTGGATTATATGGG  S: TTTTTTTTTTACATCTGTGGTATATCATTACA |
| 43 |  | *TAB2* | 6 | rs521845  (T/G) | F: TACCGCTAATGGCTACTTTGG  R: CAGGAGTTCAAGGATGTAGTG  S: TTTTTTTTTTTTTTTTTTTTCTAGGGCGGTTGAGAAG TGAAC |
| 44 | Other | *Notch1* | 9 | rs3124603  (C/T) | F: ACTCGTTGATGTTGGTCTCG  R: ATCCTGAGTAGGTGGGAATG  S: TTTTTTTTTTTTTCGTCCCCGTGTACCCTGGACCG |
| 45 |  | *AUTS2* | 7 | rs35075890  (A/G) | F: TTTGAAACCATGACCTTGGGC  R: CACTTCTGCCCATTGTTCTAC  S: GTGAAGGGCTATCACAGCACAC |
| 46 |  | *PTPRN2* | 7 | rs10081191  (C/A) | F: GAGGGAAAACAGAGAGAATGC  R: TTCCTCATGTACCAGCATCAG  S: TTCCTAGGACAGGCTGACCCCA |
| 47 |  | *SIK3* | 11 | rs493134  (T/C) | F: TATGTGCCCTCTTTCTCTTGG  R: CAGTAAACCAGTCAGTTGCTC  S: TTTTTTTTTTTTTTTTTTTTATGTAGCCTGTTGGCATT ATGT |
| 48 |  | *SIK3* | 11 | rs6589574  (G/A) | F: GGGCCCCTAAGAATTTGTGT  R: CCACCACACCCAGCTAATTT  S: GCAGTGCTTCTTGAACTTTCAC |
| 49 |  | *SIK3* | 11 | rs7121898  (A/T) | F: AAAAGCTAGGTGATGCTCCAG  R: TCCGGTATAGCATCGTGTATC  S: TTTTTTTTTTTTTTTTTTTTTTTACTAAGGAAGAAAAT CCTTAGA |
| 50 |  | *OTOG* | 11 | rs7106021  (G/A) | F: GTGCAAAGTGTGTGGATGTATG  R: ATCAGAAAGCTGAGTCTACCC  S: GTATTCACATGTGAGTGTAAAT |
| 51 |  | *XPO5* | 6 | rs11077  (A/C) | F: TTTGGGCAAGAATCTGGTCAC  R: ACAGATGTAGCCTAGTTGGTC  S: TTTTTTTTTTACCTCCAAGGACCAGGGCTGGG |
| 52 |  | *GAPDH* | 12 | rs6489721  (T/C) | F: GCCTTTGAAAGAAAGAAAGGG  R: TGGGCACGCACCGAGCGTGTGG  S: TTTTTTTTTTATGAAACAGGAGGACTTTGGGA |
| 53 |  | *JNK1* | 10 | rs11598320  (T/A) | F: TTCCCTCTCCTTTCCTCTAGAC  R: TATCCCAATCCACGGACAATC  S: TTTTTTTTTTCACTTTTTAGGGAATGCTGAGA |
| 54 |  | *JNK1* | 10 | rs8428  (T/C) | F: CAATGTGGATGTCGAGTAGTG  R: CTCCTGTTGGTCACAAATTCC  S: AACTGTTTACATTTTCTATCTG |
| 55 |  | *AKT2* | 19 | rs2304186  (G/T) | F: GGAGGTGGAGTCTTCCAAATG  R: ACTTCCCCCAGTTCTCCTACTC  S: TTTTTTTTTTTTTTTTTTAGGCTAAGTAAAAAGTTAG GGG |
| 56 |  | *AKT2* | 19 | rs41275750  (C/G) | F: ATTCTGGGCTACCCAGATCTTC  R: TTTGCCTTGGTGATGGAGTCAG  S: TTTTTTTTTTTTTTTTTTTTTTTTTTCCAGCCTGAGCT CCTGCACACA |
| 57 |  | *AKT2* | 19 | rs76524493  (I/D) | F: TTGTAGAGATGAAGGTCTCCC  R: TGAGCATGTGTGGTTATCCTG  S: TTTTTTTTTTTTTTTTTTTTTTTTTTATCTTACCAAAT AAATTTTGAG |
| 58 |  | *MYO1A* | 12 | rs1552245  (G/A) | F: AGTTCCAGGCACTAAGTAGAC  R: CATGATCACTGTGTGCCTTAG  S: CCCATAGCAATTTGCCTACACT |
| 59 |  | *STAT3* | 17 | rs1053023  (A/G) | F: AGCTGATCAGAGTTTCTGTGG  R: CCACCTTATAGGTAGGTAAGC  S: GAGCTGAGCCCTGTTGTGGCCC |
| 60 |  | *STAT3* | 17 | rs1053005  (A/G) | F: AGCTGATCAGAGTTTCTGTGG  R: CCACCTTATAGGTAGGTAAGC  S: TTTTTTTTTTTTTTTTTTTTAAGGGGGAGACGACCTT CTCTA |

|  |
| --- |

Chr.: chromosome.

A: the major allele; B: the minor allele.

F: forward primer; R: reverse primer; S: SNaPshot primer.
