# Supplementary material for: Interactions of genetic variations in FAS, GJB2 and PTPRN2 are associated with noise-induced hearing loss: a case-control study in China
Source: BMC Med Genomics. 2024 Jan 11;17:18. doi: 10.1186/s12920-023-01790-7 (PMC10785407; doi:10.1186/s12920-023-01790-7)
Supplement: Supplementary file 2 — Supplementary Table 2: General demographic characteristics of subjects in screening stage [file 12920_2023_1790_MOESM2_ESM.docx]

**Supplementary Table 2.** General demographic characteristics of subjects in screening stage.

| **Characteristics** | **Total**  **(n=166)** | **NIHL**  **(n=83)** | **Control**  **(n=83)** | ***t / χ^2^*** | ***p^a^*** |
| --- | --- | --- | --- | --- | --- |
| Age (Mean±SD, years old) | 48.32±5.69 | 48.46±5.42 | 48.17±5.99 | 0.327 | 0.744 |
| Years of Exposure-Noise  (Mean±SD, years) | 12.24±5.75 | 12.37±5.40 | 12.10±6.11 | 0.306 | 0.760 |
| CNE [Mean±SD, dB(A)·year)] | 96.51±5.49 | 96.54±5.66 | 96.48±5.32 | 0.075 | 0.941 |
| BHFTA [Mean±SD, dB] | 41.83±16.35 | 55.23±10.38 | 28.02±7.49 | 19.368 | <0.001^*^ |
| Sex [n(%)] |  |  |  | 2.473 | 0.116 |
| Male | 143 (86.1) | 75 (90.4) | 68 (81.9) |  |  |
| Female | 23 (13.9) | 8 (9.6) | 15 (18.1) |  |  |
| Nationality [n(%)] |  |  |  | 0.312 | 0.576 |
| Han | 152 (91.6) | 77 (92.8) | 75 (90.4) |  |  |
| Non-Han | 14 (8.4) | 6 (7.2) | 8 (9.6) |  |  |
| Time of Wearing PPE [n(%)] |  |  |  | 2.420 | 0.490 |
| never | 22 (13.3) | 15 (18.1) | 13 (15.7) |  |  |
| Less than half of working hours | 25 (15.1) | 42 (50.6) | 53 (63.9) |  |  |
| More than half of working hours | 117 (70.5) | 24 (28.9) | 8 (9.6) |  |  |
| Smoking [n(%)] |  |  |  | 3.274 | 0.195 |
| Yes | 71 (42.8) | 39 (47.0) | 32 (38.6) |  |  |
| No | 81 (48.8) | 40 (48.2) | 41 (49.4) |  |  |
| Drinking [n(%)] |  |  |  | 4.274 | 0.118 |
| Yes | 56 (33.7) | 28 (33.7) | 28 (33.7) |  |  |
| No | 79 (47.6) | 52 (62.7) | 45 (54.2) |  |  |

a: Two-sided *χ^2^* test was used for comparing the frequency distribution and two-sided *t*-test was used for comparing the mean values of the continuous variables.

*: *p* < 0.05.
