# Supplementary material for: Interactions of genetic variations in FAS, GJB2 and PTPRN2 are associated with noise-induced hearing loss: a case-control study in China
Source: BMC Med Genomics. 2024 Jan 11;17:18. doi: 10.1186/s12920-023-01790-7 (PMC10785407; doi:10.1186/s12920-023-01790-7)
Supplement: Supplementary file 3 — Supplementary Table 3: Distribution of genotypes of the selected SNPs and the results of Hardy-Weinberg test [file 12920_2023_1790_MOESM3_ESM.docx]

**Supplementary Table 3.** Distribution of genotypes of the selected SNPs and the results of Hardy-Weinberg test.

| **Code** | **Gene** | **SNPs ID** | **Genotype frequency (AA/AB/BB)** | | ***p^a^*** | ***p^b^*** | ***p^c^*** | ***p^d^*** | ***p^e^*** | ***p_HWE_*** |
| --- | --- | --- | --- | --- | --- | --- | --- | --- | --- | --- |
|  |  |  | **NIHL (n=83)** | **Control (n=83)** |  |  |  |  |  |  |
| 1 | *AKT2* | rs76524493 | 72/11/0  (86.7/13.3/0.0) | 75/8/0  (90.4/9.6/0.0) | / | 0.465 | 0.465 | / | 0.478 | 1.000 |
| 2 | *AKT2* | rs2304186 | 21/43/10  (25.3/51.8/22.9) | 19/48/16  (22.9/57.8/19.3) | 0.568 | 0.717 | 0.436 | 0.877 | 0.913 | 0.192 |
| 3 | *AKT2* | rs41275750 | 71/11/1  (85.5/13.3/1.2) | 74/8/1  (89.2/9.6/1.2) | 1.000 | 0.484 | 0.465 | 0.977 | 0.517 | 0.275 |
| 4 | *APEX1* | rs1130409 | 13/41/29  (15.7/49.4/34.9) | 15/38/30  (18.1/45.8/36.1) | 0.871 | 0.678 | 0.641 | 0.812 | 0.911 | 0.659 |
| 5 | *APE1* | rs1760944 | 19/32/32  (22.9/38.6/38.6) | 16/37/30  (19.3/44.6/36.1) | 0.748 | 0.568 | 0.431 | 0.800 | 0.911 | 0.500 |
| 6 | *AUTS2* | rs35075890 | 71/11/1  (85.5/13.3/1.2) | 64/19/0  (77.1/22.9/0.0) | 0.316 | 0.163 | 0.107 | 1.000 | 0.265 | 0.059^#^ |
| 7 | *CARD8* | rs2043211 | 23/32/28 (27.7/38.6/33.7) | 20/42/21  (24.1/50.6/25.3) | 0.234 | 0.595 | 0.118 | 0.725 | 0.660 | 1.000 |
| 8 | *CASP3* | rs6948 | 49/30/4  (59.0/36.1/4.8) | 53/29/1  (63.9/34.9/1.2) | 0.173 | 0.524 | 0.871 | 0.163 | 0.344 | 0.133 |
| 9 | *CASP3* | rs1049216 | 4/31/48  (4.8/37.3/57.8) | 1/30/52  (1.2/36.1/62.7) | 0.526 | 0.364 | 0.872 | 0.348 | 0.349 | 0.185 |
| 10 | *CASP7* | rs2227310 | 35/38/10  (42.2/45.8/12.0) | 30/38/15  (36.1/45.8/18.1) | 0.278 | 0.427 | 1.000 | 0.239 | 0.258 | 0.658 |
| 11 | *CASP7* | rs4353229 | 10/37/36  (12.0/44.6/43.4) | 15/38/30  (18.1/45.8/36.1) | 0.341 | 0.278 | 0.876 | 0.215 | 0.213 | 0.656 |
| 12 | *CAT* | rs7943316 | 38/38/7  (45.8/45.8/8.4) | 37/41/5  (44.6/49.4/6.0) | 0.549 | 0.876 | 0.641 | 0.622 | 0.906 | 0.115 |
| 13 | *CAT* | rs769217 | 24/42/17  (28.9/50.6/20.5) | 24/49/10  (28.9/59.0/12.0) | 0.141 | 1.000 | 0.275 | 0.279 | 0.439 | 0.039^*^ |
| 14 | *CAT* | rs769214 | 7/37/39  (8.4/44.6/47.0) | 5/42/36  (6.0/50.6/43.4) | 0.640 | 0.549 | 0.437 | 0.683 | 0.906 | 0.133 |
| 15 | *CDH23* | rs4128133 | 83/0/0  (100.0/0.0/0.0) | 83/0/0  (100.0/0.0/0.0) | / | / | / | / | / | / |
| 16 | *CDH23* | rs2394795 | 21/49/13  (25.3/59.0/15.7) | 16/43/24  (19.3/51.8/28.9) | 0.040^*^ | 0.351 | 0.349 | 0.062^#^ | 0.079^#^ | 0.660 |
| 17 | *CDH23* | rs3752752 | 20/37/26  (24.1/44.6/31.3) | 17/45/21  (20.5/54.2/25.3) | 0.389 | 0.576 | 0.214 | 0.908 | 0.826 | 0.374 |
| 18 | *FAS* | rs1468063 | 28/29/26  (33.7/34.9/31.3) | 26/44/13  (31.3/53.0/15.7) | 0.017^*^ | 0.740 | 0.019^*^ | 0.153 | 0.225 | 0.507 |
| 19 | *FAS* | rs2862833 | 28/30/25  (33.7/36.1/30.1) | 14/43/26  (16.9/51.8/31.3) | 0.866 | 0.012^*^ | 0.042^*^ | 0.087^#^ | 0.099^#^ | 0.506 |
| 20 | *FOXO3* | rs2802292 | 51/23/9  (61.4/27.7/10.8) | 42/35/6  (50.6/42.2/7.2) | 0.417 | 0.351 | 0.051^#^ | 0.709 | 0.456 | 0.581 |
| 21 | *FOXO3* | rs10457180 | 50/23/10  (60.2/27.7/12.0) | 43/34/6  (51.8/41.0/7.2) | 0.293 | 0.576 | 0.072^#^ | 0.516 | 0.710 | 1.000 |
| 22 | *GAPDH* | rs6489721 | 32/37/14  (38.6/44.6/16.9) | 26/43/14  (31.3/51.8/16.9) | 1.000 | 0.079^#^ | 0.351 | 0.652 | 0.503 | 0.491 |
| 23 | *GJB2* | rs3751385 | 25/26/32  (30.1/31.3/38.6) | 28/38/17  (33.7/45.8/20.5) | 0.011^*^ | 0.617 | 0.056^#^ | 0.065^#^ | 0.048^*^ | 0.655 |
| 24 | *GRHL2* | rs666026 | 43/30/10  (51.8/36.1/12.0) | 44/29/10  (53.0/34.9/12.0) | 1.000 | 0.876 | 0.871 | 0.963 | 0.905 | 0.184 |
| 25 | *HDAC2* | rs10499080 | 28/41/14  (33.7/49.4/16.9) | 18/49/16  (21.7/59.0/19.3) | 0.687 | 0.083^#^ | 0.213 | 0.224 | 0.186 | 0.081^#^ |
| 26 | *HOTAIR* | rs874945 | 54/29/0  (65.1/34.9/0.0) | 56/22/5  (67.5/26.5/6.0) | 0.023^*^ | 0.743 | 0.239 | 0.090^#^ | 0.671 | 0.277 |
| 27 | *hOGG1* | rs1052133 | 17/44/22  (20.5/53.0/26.5) | 13/37/33  (15.7/44.6/39.8) | 0.070^#^ | 0.420 | 0.277 | 0.141 | 0.096^#^ | 0.640 |
| 28 | *JNK1* | rs11598320 | 50/31/2  (60.2/37.3/2.4) | 53/25/5  (63.9/30.1/6.0) | 0.247 | 0.631 | 0.325 | 0.527 | 1.000 | 0.503 |
| 29 | *JNK1* | rs8428 | 34/44/5  (41.0/53.0/6.0) | 36/37/10  (43.4/44.6/12.0) | 0.176 | 0.753 | 0.277 | 0.282 | 0.727 | 1.000 |
| 30 | *KCNE1* | rs1805127 | 43/35/5  (51.8/42.2/6.0) | 41/37/5  (49.4/44.6/6.0) | 1.000 | 0.756 | 0.754 | 1.000 | 0.806 | 0.268 |
| 31 | *KCNE1* | rs3453 | 13/42/28  (15.7/50.6/33.7) | 11/49/23  (13.3/59.0/27.7) | 0.400 | 0.659 | 0.275 | 0.952 | 0.739 | 0.044^*^ |
| 32 | *KCNE1* | rs1805128 | 83/0/0  (100.0/0.0/0.0) | 83/0/0  (100.0/0.0/0.0) | / | / | / | / | / | / |
| 33 | *KCNMA1* | rs696211 | 39/38/6  (47.0/45.8/7.2) | 41/34/8  (49.4/41.0/9.6) | 0.576 | 0.756 | 0.531 | 0.684 | 1.000 | 1.000 |
| 34 | *KCNMA1* | rs7910544 | 70/12/1  (84.3/14.5/1.2) | 63/18/2  (75.9/21.7/2.4) | 0.560 | 0.173 | 0.226 | 0.938 | 0.158 | 0.634 |
| 35 | *KCNQ4* | rs4660468 | 20/32/31  (24.1/38.6/37.3) | 18/38/27  (21.7/45.8/32.5) | 0.515 | 0.712 | 0.346 | 0.937 | 0.825 | 0.519 |
| 36 | *NOX3* | rs12195525 | 71/12/0  (85.5/14.5/0.0) | 67/16/0  (80.7/19.3/0.0) | / | 0.407 | 0.407 | / | 0.430 | 0.599 |
| 37 | *Notch1* | rs3124603 | 60/20/3  (72.3/24.1/3.6) | 64/18/1  (77.1/21.7/1.2) | 0.311 | 0.475 | 0.712 | 0.589 | 0.341 | 1.000 |
| 38 | *NRF2* | rs77684420 | 65/17/1  (78.3/20.5/1.2) | 62/19/2  (74.7/22.9/2.4) | 0.560 | 0.583 | 0.706 | 0.541 | 0.509 | 1.000 |
| 39 | *NRF2* | rs6726395 | 37/33/13  (44.6/39.8/15.7) | 42/27/14  (50.6/32.5/16.9) | 0.833 | 0.437 | 0.332 | 0.906 | 0.644 | 0.023^*^ |
| 40 | *MYH14* | rs588035 | 72/11/0  (86.7/13.3/0.0) | 69/14//0  (83.1/16.9/0.0) | / | 0.515 | 0.515 | / | 0.533 | 0.637 |
| 41 | *MYH14* | rs667907 | 47/30/6  (56.6/36.1/7.2) | 51/27/5  (61.4/32.5/6.0) | 0.755 | 0.528 | 0.624 | 0.679 | 0.519 | 0.754 |
| 42 | *MYO1A* | rs1552245 | 46/34/3  (55.4/41.0/3.6) | 52/28/3  (62.7/33.7/3.6) | 1.000 | 0.344 | 0.336 | 1.000 | 0.429 | 1.000 |
| 43 | *OTOG* | rs7106021 | 68/13/2  (81.9/15.7/2.4) | 73/9/1  (88.0/10.8/1.2) | 0.560 | 0.278 | 0.360 | 0.961 | 0.236 | 0.285 |
| 44 | *PCDH15* | rs1104085 | 81/2/0  (97.6/2.4/0.0) | 83/0/0  (100.0/0.0/0.0) | / | 0.477 | 0.477 | / | 0.156 | / |
| 45 | *PCDH15* | rs7095441 | 83/0/0  (100.0/0.0/0.0) | 83/0/0  (100.0/0.0/0.0) | / | / | / | / | / | / |
| 46 | *PMCA2* | rs3209637 | 25/38/20  (30.1/45.8/24.1) | 17/41/25  (20.5/49.4/30.1) | 0.383 | 0.153 | 0.641 | 0.160 | 0.154 | 1.000 |
| 47 | *PON3* | rs11767787 | 2/22/59  (2.4/26.5/71.1) | 1/30/52  (1.2/36.1/62.7) | 0.248 | 1.000 | 0.181 | 1.000 | 0.386 | 0.183 |
| 48 | *PON3* | rs17882539 | 59/23/1  (71.1/27.7/1.2) | 53/29/1  (63.9/34.9/1.2) | 1.000 | 0.320 | 0.315 | 1.000 | 0.379 | 0.141 |
| 49 | *PON3* | rs13226149 | 59/23/1  (71.1/27.7/1.2) | 52/30/1  (62.7/36.1/1.2) | 1.000 | 0.248 | 0.244 | 1.000 | 0.308 | 0.176 |
| 50 | *POU4F3* | rs891969 | 54/28/1  (65.1/33.7/1.2) | 55/25/3  (66.3/30.1/3.6) | 0.311 | 0.870 | 0.617 | 0.649 | 0.887 | 1.000 |
| 51 | *PTPRN2* | rs10081191 | 44/30/9  (53.0/36.1/10.8) | 36/42/5  (43.4/50.6/6.0) | 0.264 | 0.214 | 0.060^#^ | 0.518 | 0.632 | 0.133 |
| 52 | *SIK3* | rs7121898 | 54/26/3  (65.1/31.3/3.6) | 54/28/1  (65.1/33.7/1.2) | 0.311 | 1.000 | 0.740 | 0.636 | 0.778 | 0.287 |
| 53 | *SIK3* | rs493134 | 22/42/19  (26.5/50.6/22.9) | 14/44/25  (16.9/53.0/30.1) | 0.291 | 0.132 | 0.756 | 0.110 | 0.124 | 0.510 |
| 54 | *SIK3* | rs6589574 | 48/30/5  (57.8/36.1/6.0) | 32/41/10  (38.6/49.4/12.0) | 0.176 | 0.013^*^ | 0.084^#^ | 0.056^#^ | 0.012^*^ | 0.481 |
| 55 | *STAT3* | rs1053023 | 31/47/5  (37.3/56.6/6.0) | 32/39/12  (38.6/47.0/14.5) | 0.073^#^ | 0.873 | 0.214 | 0.145 | 0.493 | 1.000 |
| 56 | *STAT3* | rs1053005 | 32/47/4  (38.6/56.6/4.8) | 32/39/12  (38.6/47.0/14.5) | 0.035^*^ | 1.000 | 0.214 | 0.072^#^ | 0.359 | 0.226 |
| 57 | *TAB2* | rs521845 | 27/45/11  (32.5/54.2/13.3) | 35/34/14  (42.2/41.0/16.9) | 0.515 | 0.199 | 0.087^#^ | 0.969 | 0.573 | 0.352 |
| 58 | *XRCC1* | rs1799782 | 48/27/8  (57.8/32.5/9.6) | 41/36/6  (49.4/43.4/7.2) | 0.576 | 0.276 | 0.150 | 0.823 | 0.538 | 0.782 |
| 59 | *WHRN* | rs12339210 | 80/3/0  (96.4/3.6/0.0) | 80/3/0  (96.4/3.6/0.0) | / | 1.000 | 1.000 | / | 1.000 | 1.000 |
| 60 | *XPO5* | rs11077 | 75/8/0  (90.4/9.6/0.0) | 73/9/1  (88.0/10.8/1.2) | 0.316 | 0.618 | 0.798 | 0.995 | 0.478 | 0.284 |

a: Two-sided *χ^2^* test under recessive model.

b: two-sided *χ^2^* test under dominant model.

c: two-sided *χ^2^* test under super-dominant model.

d: two-sided *χ^2^* test under homozygote model.

e: two-sided *χ^2^* test under the allele model.

AA: wild genotype; AB: heterozygous mutation genotype; BB: homozygous mutant genotype.

*: *p* < 0.05; #: 0.05 ≤ *p* < 0.10; *p_HWE_*: *p*-values of deviation from HWE of the control group.
