# Supplementary material for: Interactions of genetic variations in FAS, GJB2 and PTPRN2 are associated with noise-induced hearing loss: a case-control study in China
Source: BMC Med Genomics. 2024 Jan 11;17:18. doi: 10.1186/s12920-023-01790-7 (PMC10785407; doi:10.1186/s12920-023-01790-7)
Supplement: Supplementary file 4 — Supplementary Table 4: General demographic characteristics of subjects in replication stage [file 12920_2023_1790_MOESM4_ESM.docx]

**Supplementary Table 4.** General demographic characteristics of subjects in replication stage.

| **Characteristics** | **Total (n=405)** | **NIHL (n=153)** | **Control (n=252)** | ***t / χ^2^*** | ***p^a^*** |
| --- | --- | --- | --- | --- | --- |
| Age (Mean±SD, years old) | 46.08±7.57 | 48.58±6.95 | 45.18±7.60 | -4.493 | <0.001^*^ |
| Years of Exposure-Noise  (Mean±SD, years) | 11.80±6.04 | 12.41±6.21 | 11.90±5.93 | -0.826 | 0.409 |
| CNE [Mean±SD, dB(A)·year)] | 97.00±6.11 | 96.35±5.44 | 97.49±6.54 | 1.849 | 0.065 |
| BHFTA [Mean±SD, dB] | 43.58±15.85 | 55.86±10.72 | 24.92±8.17 | -30.560 | <0.001^*^ |
| Sex [n(%)] |  |  |  | 1.190 | 0.275 |
| Male | 359 (88.6) | 139 (90.8) | 220 (87.3) |  |  |
| Female | 46 (11.4) | 14 (9.2) | 32 (12.7) |  |  |
| Nationality [n(%)] |  |  |  | 0.625 | 0.732 |
| Han | 381 (94.1) | 114 (94.1) | 237 (94.0) |  |  |
| Non-Han | 23 (5.7) | 9 (5.9) | 14 (5.6) |  |  |
| Time of Wearing PPE [n(%)] |  |  |  | 10.515 | 0.015^*^ |
| never | 52 (12.8) | 28 (18.3) | 24 (9.5) |  |  |
| Less than half of working hours | 62 (15.3) | 22 (14.4) | 40 (15.9) |  |  |
| More than half of working hours | 284 (70.1) | 98 (64.1) | 186 (73.8) |  |  |
| Smoking [n(%)] |  |  |  | 1.129 | 0.569 |
| Yes | 181 (44.7) | 72 (47.1) | 109 (43.3) |  |  |
| No | 206 (50.9) | 73 (47.7) | 133 (52.8) |  |  |
| Drinking [n(%)] |  |  |  | 8.424 | 0.015^*^ |
| Yes | 172 (42.5) | 51 (33.3) | 121 (48.0) |  |  |
| No | 214 (52.8) | 94 (61.4) | 120 (47.6) |  |  |

a: Two-sided *χ^2^* test was used for comparing the frequency distribution and two-sided *t*-test was used for comparing the mean values of the continuous variables.

*: *p* < 0.05.
