# Supplementary material for: Interactions of genetic variations in FAS, GJB2 and PTPRN2 are associated with noise-induced hearing loss: a case-control study in China
Source: BMC Med Genomics. 2024 Jan 11;17:18. doi: 10.1186/s12920-023-01790-7 (PMC10785407; doi:10.1186/s12920-023-01790-7)
Supplement: Supplementary file 5 — Supplementary Table 5: Distribution of genotypes of 8 NIHL-associated SNPs and the results of Hardy-Weinberg test [file 12920_2023_1790_MOESM5_ESM.docx]

**Supplementary Table 5.** Distribution of genotypes of 8 NIHL-associated SNPs and the results of Hardy-Weinberg test.

| **Code** | **Gene** | **SNPs ID** | **Genotype frequency (AA/AB/BB)** | | ***p^a^*** | ***p^b^*** | ***p^c^*** | ***p^d^*** | ***p^e^*** | ***p_HWE_*** |
| --- | --- | --- | --- | --- | --- | --- | --- | --- | --- | --- |
|  |  |  | **NIHL (n=153)** | **Control (n=252)** |  |  |  |  |  |  |
| 1 | *CDH23* | rs2394795 | 38/92/23  (24.9/60.1/15.0) | 44/136/72  (17.5/54.0/28.5) | 0.002^*^ | 0.073^#^ | 0.225 | 0.002^*^ | 0.005^*^ | 0.154 |
| 2 | *FAS* | rs1468063 | 64/51/38  (41.8/33.3/24.9) | 93/121/38  (36.9/48.0/15.1) | 0.015^*^ | 0.324 | 0.004^*^ | 0.183 | 0.437 | 0.893 |
| 3 | *FAS* | rs2862833 | 41/54/58  (26.8/35.3/37.9) | 46/121/85  (18.3/48.0/33.7) | 0.394 | 0.042^*^ | 0.012^*^ | 0.329 | 0.442 | 0.795 |
| 4 | *GJB2* | rs3751385 | 37/59/57  (24.2/38.6/37.2) | 89/117/46  (35.3/46.4/18.3) | <0.001^*^ | 0.019^*^ | 0.122 | <0.001^*^ | <0.001^*^ | 0.518 |
| 5 | *PTPRN2* | rs10081191 | 80/57/16  (52.3/37.3/10.4) | 112/116/24  (44.4/46.0/9.6) | 0.760 | 0.125 | 0.083^#^ | 0.846 | 0.304 | 0.474 |
| 6 | *SIK3* | rs6589574 | 81/57/15  (52.9/37.3/9.8) | 106/116/30  (42.1/46.0/11.9) | 0.514 | 0.033^*^ | 0.083^#^ | 0.222 | 0.056^#^ | 0.891 |
| 7 | *STAT3* | rs1053023 | 61/77/15  (39.9/50.3/9.8) | 110/105/37  (43.7/41.7/14.6) | 0.155 | 0.455 | 0.089^#^ | 0.363 | 0.874 | 0.176 |
| 8 | *STAT3* | rs1053005 | 61/77/15  (39.9/50.3/9.8) | 110/105/37  (43.7/41.7/14.6) | 0.155 | 0.455 | 0.089^#^ | 0.363 | 0.874 | 0.176 |

a: Two-sided *χ^2^* test under recessive model.

b: two-sided *χ^2^* test under dominant model.

c: two-sided *χ^2^* test under super-dominant model.

d: two-sided *χ^2^* test under homozygote model.

e: two-sided *χ^2^* test under the allele model.

AA: wild genotype; AB: heterozygous mutation genotype; BB: homozygous mutant genotype.

*: *p* < 0.05; #: 0.05 ≤ *p* < 0.10; *p_HWE_*: *p*-values of deviation from HWE of the control group.
